# A review on biosynthesis of silver nanoparticles and their biocidal properties

**DOI:** 10.1186/s12951-018-0334-5

**Published:** 2018-02-16

**Authors:** Khwaja Salahuddin Siddiqi, Azamal Husen, Rifaqat A. K. Rao

**Affiliations:** 1 Department of Chemistry, Aligarh Muslim University, Aligarh, Uttar Pradesh 202002 India; 2 Department of Biology, College of Natural and Computational Sciences, University of Gondar, P.O. Box # 196, Gondar, Ethiopia; 3 Department of Applied Chemistry, Zakir Husain College of Engineering and Technology, Aligarh Muslim University, Aligarh, Uttar Pradesh 202002 India

**Keywords:** Silver nanoparticles, Antimicrobial activity, Antioxidant activity, Green synthesis, Toxicity mechanism

## Abstract

Use of silver and silver salts is as old as human civilization but the fabrication of silver nanoparticles (Ag NPs) has only recently been recognized. They have been specifically used in agriculture and medicine as antibacterial, antifungal and antioxidants. It has been demonstrated that Ag NPs arrest the growth and multiplication of many bacteria such as *Bacillus cereus*, *Staphylococcus aureus*, *Citrobacter koseri*, *Salmonella typhii*, *Pseudomonas aeruginosa*, *Escherichia coli*, *Klebsiella pneumonia, Vibrio parahaemolyticus* and fungus *Candida albicans* by binding Ag/Ag^+^ with the biomolecules present in the microbial cells. It has been suggested that Ag NPs produce reactive oxygen species and free radicals which cause apoptosis leading to cell death preventing their replication. Since Ag NPs are smaller than the microorganisms, they diffuse into cell and rupture the cell wall which has been shown from SEM and TEM images of the suspension containing nanoparticles and pathogens. It has also been shown that smaller nanoparticles are more toxic than the bigger ones. Ag NPs are also used in packaging to prevent damage of food products by pathogens. The toxicity of Ag NPs is dependent on the size, concentration, pH of the medium and exposure time to pathogens.

## Introduction

Nanoparticles exhibit novel properties which depend on their size, shape and morphology which enable them to interact with plants, animals and microbes [1–7]. Silver nanoparticles (Ag NPs) have shown excellent bactericidal properties against a wide range of microorganisms [8–11]. They are prepared from different perspectives, often to study their morphology or physical characteristics. Some authors have used chemical method [12] and mistaken it with green synthesis, although they have done it inadvertently. The Ag NPs and their application in electronics, catalysis, drugs and in controlling microorganism development in biological system have made them eco-friendly [1, 8, 9, 13]. Biogenic synthesis of Ag NPs involves bacteria, fungi, yeast, actinomycetes and plant extracts [1, 10, 11, 13–15]. Recently, a number of parts of plants such as flowers, leaves and fruits [1], besides enzymes, have been used for the synthesis of gold and silver nanoparticles. The size, morphology and stability of nanoparticles depend on the method of preparation, nature of solvent, concentration, strength of reducing agent and temperature [1, 6, 10, 11].

Of all the nanoparticles developed and characterized thus far, Ag NPs assume a significant position owing to their inherent characteristic of acting as an antimicrobial agent even in solid state. Although, its significance was recognized much earlier, it was not well exploited except for its use in oriental medicine and in coins. It is estimated that nearly 320 tons of Ag NPs are manufactured every year and used in nanomedical imaging, biosensing and food products [16, 17].

There is a continuous increase in the number of multi-drug resistant bacterial and viral strains due to mutation, pollution and changing environmental conditions. To circumvent this predicament scientists are trying to develop drugs for the treatment of such microbial infections. Many metal salts and metal nanoparticles have been found to be effective in inhibiting the growth of many infectious bacteria. Silver and Ag NPs occupy a prominent place in the series of such metals which are used as antimicrobial agents from time immemorial [18, 19]. Silver salts are used to inhibit the growth of a variety of bacteria in human system. They are used in catheters, cuts, burns and wounds to protect them against infection [20, 21]. Das et al. [22] have reported that small sized Ag NPs are excellent growth inhibitors of certain bacteria. Ag NPs synthesized from silk sericin (SS), a water-soluble protein extracted from silk worms at pH 11, contain hydrophilic proteins with highly polar groups like hydroxyl, carboxyl and amino functional groups. Molecules containing the above functional groups act as reducing agents for AgNO_3_ to produce elemental silver. Aramwit et al. [23] have suggested that the hydroxyl groups of SS are supposed to form complex with silver ions and prevent their aggregation or precipitation [24, 25]. Ag NPs in elemental state may be segregated due to large molecules present in the solvent but may not be complexed as both of them are neutral. The antibacterial activity of SS-capped Ag NPs against gram positive and gram negative bacteria has been screened. It was found that MIC falls between 0.001 and 0.008 mM for both types of microorganisms namely *Staphylococcus aureus*, *Bacillus subtilis*, *Pseudomonas aeruginosa*, *Acinetobacter baumannii* and *Escherichia coli.*

Although, several reviews have been published on the fabrication and characterization of silver nanoparticles, very limited reports are available on their green synthesis, biocidal properties and mechanism of action [8, 9, 13, 16, 23]. Thus, in this review, we have attempted to give a comprehensive detail of the biosynthesis of Ag NPs from herbal extracts, fungi and bacteria. Their potential as antimicrobial agent and the mechanism of their action has also been discussed.

## Synthesis and characterization of silver nanoparticles

In general, metallic nanoparticles are produced by two methods, i.e. “bottom-up” (buildup of a material from the bottom: atom by atom, molecule by molecule or cluster by cluster) and “top-down” (slicing or successive cutting of a bulk material to get nano-sized particle) [1]. The “bottom-up” approach is usually a superior choice for the nanoparticles preparation involving a homogeneous system wherein catalysts (for instance, reducing agent and enzymes) synthesize nanostructures that are controlled by the catalyst itself. However, the “top-down” approach generally works with the material in its bulk form, and the size reduction to nanoscale is achieved by specialized ablations, for instance thermal decomposition, mechanical grinding, etching, cutting, and sputtering. The main demerit of the top-down approach is the surface structural defects. Such defects have significant impact on the physical features and surface chemistry of metallic nanoparticles. Several methodologies are available for the synthesis of Ag NPs namely, chemical methods [26–29]; physical methods [30–32] and biological methods [1, 10, 11]. Chemical method of synthesis can be subdivided into chemical reduction, electrochemical, irradiation-assisted chemical and pyrolysis methods [33]. Ag NPs synthesis in solution requires metal precursor, reducing agents and stabilizing or capping agent. Commonly used reducing agents are ascorbic acid, alcohol, borohydride, sodium citrate and hydrazine compounds. Sotiriou and Pratsinis [28] have shown that the Ag NPs supported on nanostructured SiO_2_ were obtained by flame aerosol technology, which allows close control of silver content and size. Also, silver/silica nanoparticles with relatively narrow size distribution were obtained by flame spray pyrolysis [29]. However, physical methods do not require lethal and highly reactive chemicals and generally have a fast processing time. These methods include arc-discharge [31], physical vapor condensation [30], energy ball milling method [34] and direct current magnetron sputtering [32]. Physical methods have another advantage over chemical methods in that the Ag NPs have a narrow size distribution [32], while the main demerits are consumption of high energy [32]. Thus, biological synthesis of Ag NPs from herbal extract and/or microorganisms has appeared as an alternative approach as these routes have several advantages over the chemical and physical methods of synthesis. It is also a well-established fact that these routes are simple, cost-effective, eco-friendly and easily scaled up for high yields and or production [1–3]. Biosynthesis of metal and metal oxide nanoparticles using biological agents such as bacteria, fungi, yeast, plant and algal extracts has gained popularity in the area of nanotechnology [1–3, 5, 6, 10, 11].

Plants and their parts contain carbohydrates, fats, proteins, nucleic acids, pigments and several types of secondary metabolites which act as reducing agents to produce nanoparticles from metal salts without producing any toxic by-product. The details have been provided in Table 1. Similarly, biomolecules such as enzymes, proteins and bio-surfactants present in microorganisms serve as reducing agents. For instance, in many bacterial strains, bio-surfactants are used as capping and/or stabilizing agents (Table 2).

**Table 1:** Plant-mediated synthesis of silver nanoparticles

Plant	Plant part	Size and shape	Phytoconstituents responsible for reduction of silver nitrate	Key references
*Aloe vera*	Leaf gel (removed skin)	5–50 nm; octahedron	Flavanones and terpenoids	Logaranjan et al. [35]
	Leaf	70.7–192.02 nm; spherical (size varies through change of times and temperatures)	Lignin, hemicellulose and pectins	Tippayawat et al. [36]
	Leaf	Size varies in accordance to different parameters; spherical	Flavonoids, terpenoids and phenols	Moosa et al. [37]
*Mangifera indica*	Seed	14 nm; spherical and hexagonal	Phenolic compounds, gallotannins and tannin	Sreekanth et al. [38]
*Erigeron bonariensis*	Leaf	13 nm; spherical	Flavonoids, steroids, glycosides, triterpenes, sugars and caffeoyl derivatives	Kumar et al. [39]
*Myristica fragrans*	Bark and seeds	Spherical, polydispersed	Secondary metabolites	Jelin et al. [40]
*Momordica charantia*	Leaf	11 nm; spherical	Momorcharins, momordenol, momordicius, momordin, momordolo, charantin, charine, cucuritanes, cucurbitns, goyaglycosides and goyasaponins	Ajitha et al. [41]
*Carambola*	Fruit	16, 13, 12 nm at pH 4, 7, 10 respectively	Polysaccharides, polyols and ascorbic acid	Chowdhury et al. [42]
*Rubus glaucus*	Fruit	12–50 nm; spherical	Phenolic groups and flavonoids	Kumar et al. [43]
*Prunus serotina*	Fruit	20–80 nm (blue LED) 40–100 nm (white solar); spherical	Chlorogenic acid, catechin, proanthocyanidin, and flavonol glycosides	Kumar et al. [44]
*Piper nigrum*	Seeds	10–60 nm; rod shaped	Polysaccharides, amino acids, alkaloids, proteins and vitamins	Mohapatra et al. [45]
*Nigella sativa*	Leaf	15 nm; spherical	Alkaloids, ascorbic acid, saponins, glycosides, amino acids, flavonoids like catechin, apigenin, gallic acid and benzoates especially vanillic acid	Amooaghaie et al. [46]
*Calotropis gigantean*	Flower	10–50 nm; spherical	–	Pavani et al. [47]
*Acmella oleracea*	Flower	2–20 nm; spherical	–	Raj et al. [48]
*Piper betle*	Leaf	48–83 nm; spherical	Allylic benzenes, phenolic, amino acids, proteins, alcoholic compounds, terpenes and terpenoids	Kamachandran et al. [49]
*Morinda tinctoria*	Leaf	80–100 nm; spherical and rod	Ascorbic acid, niacin, copper and iron	Vennila and Prabha [50]
*Trigonella foenum*-*graecum*	Seeds	20–50 nm; spherical	Saponins and alkaloids	Meena and Chouhan [51]
*Picrasma quassioides*	Bark	17.5–66.5 nm; spherical	–	Sreekanth et al. [52]
*Rosa* ‘Andeli’	Petals	0.5–1.4 nm; spherical	Polyphenols and flavonoids	Suarez-Cerda et al. [53]
*Salvadora persica*	Stem	1–6 nm; spherical	Phenolic compounds	Tahir et al. [54]
*Artemisia absinthium*		5–20 nm; round shaped	Phenolic compounds and flavonoids	Ali et al. [55]
*Chelidonium majus*	Aerial parts	DLS-253.3 nm; spherical, quasi-spherical	Flavonoids and alkaloids	Barbinta-Patrascu et al. [56]
*Calotropis procera*	Flower	35 nm; face centered cubic	Tannins, triterpenes, flavonoids, steroids, alkaloids and cardiac glycosides	Babu and Prabu [57]
*Sterculia acuminata*	Fruit	~ 10 nm; spherical	Ascorbic acid, gallic acid, phenolic compounds, pyrogallol, methyl gallate and polyphenolic compounds	Bogireddy et al. [58]
*Terminalia cuneata*	Bark	25–50 nm; spherical	Tannins, saponins, triterpenoids, flavonoids, gallic acid, ellagic acid and phytosterols	Edison et al. [59]
*Cirsium japonicum*	Plant	4–8 nm; spherical	Saponins, proteins and flavonoids	Khan et al. [60]
*Isatis tinctoria*	Plant	10–15 nm; spherical	Saponins and flavonoids	Ahmad et al. [61]
*Aegle marmelos*	Fruit	22.5 nm; spherical, hexagonal, roughly circular	Phytosterols, flavonoids, alkaloids, triterpenoids and amino acids	Velmurugan et al. [62]
*Trachyspermum ammi*	Seeds	36 nm; cubic	Fatty acids, proteins, flavonoids and alkaloids	Chouhanand Meena [63]
*Eucalyptus globulus*	Leaf	1.9–4.3 and 5–25 nm with and without microwave treatment respectively	Alkaloids and flavonoids	Ali et al. [64]
*Cydonia oblonga*	Seeds	38 nm; face-centered cubic	Flavonones, terpenoids, proteins and amino acids	Zia et al. [65]
*Hydrocotyle asiatica*	Leaf	21 nm; spherical	Flavonoids and glycosides	Devi et al. [66]
*Lantana camara*	Leaf	33.8 nm; spherical	Flavonoids, proteins, saccharides secondary metabolites like alkaloids, tannins, saponins, carbohydrates, steroids and triterpenoids	Manjamadha and Muthukumar [67]
*Nyctanthes arbor*-*tristis*	Seeds	50–80 nm; spherical	Carbohydrates and phenolic compounds	Basu et al. [68]
*Pennyroyal* sp.	Leaf	19.14 ± 9.791 nm; spherical	–	Sedaghat et al. [69]
*Saraca indica*	Leaf	23 ± 2 nm; spherical	Flavonoids and steroids	Perugu et al. [70]
*Terminalia chebula*	Fruit	30 nm; distorted spherical	–	Edison et al. [71]
*Euphorbia amygdaloides*	Plant	7–20 nm; spherical	–	Cicek et al. [72]
*Pedalium murex*	Leaf	50 nm; spherical	Flavonoids, alkaloids, steroids, rosins, saponins and proteins	Anandalakshmi et al. [73]
*Chelidonium majus*	Root	15.42 nm; spherical	–	Alishah et al. [74]
*Salacia chinensis*	Powdered plant	20–80 nm; spherical, rods, triangular, hexagonal	Flavonoids, saponins, proteins, carbohydrates and phenolics	Jadhav et al. [75]
*Tamarindus indica*	Seed coat	~ 12.73 nm	Flavonoids, tannin and saponins,	Ramamurthi et al. [76]
*Parkia roxburghii*	Leaf	5–25 nm; poly dispersped, quasi-spherical	Proteins	Paul et al. [77]
*Aristolochia indica*	Leaf	32–55 nm; spherical	–	Shanmugam et al. [78]
*Cerasus serrulata*	Leaf	10–50 nm; spherical	Alcohol and phenolic compounds and proteins	Karthik et al. [79]
*Matricaria camomilia*	Flower	8–35 nm; spherical	Terpenoids, flavones and polysaccharides	Parlinska-Wojtan et al. [80]
	Flower	~ 5.5 nm; spherical	Phenolics, carbonyl and amines or alcohol groups	Ocsoy et al. [81]
	Fruits	~ 15.4 nm; spherical	Phenolics, flavonoids, terpenoids and vitamins	Swamy et al. [82]
*Alpinia calcarata*	Root	5–15 nm; quasi-spherical	Proteins, flavonoids and polyphenols	Pugazhendhi et al. [83]
*Salvinia molesta*	Leaf	12.46 nm; spherical	Alkaloids, flavonoids, tannins, phenols, sugars and proteins	Verma et al. [84]
*Helicteres isora*	Root	16–95 nm; spherical	Steroids, terpenoids, alkaloids, carbohydrates and phenolic compounds	Bhakya et al. [85]
*Mukia maderaspatana*	Leaf	158 nm; spherical	Phenolic compounds	Harshiny et al. [86]
*Ficus benghalensis* and *Azadirachta indica*	Bark	60 nm; spherical	Flavonoids, terpenoids and phenols	Nayak et al. [87]
*Azadirachta indica*	Leaf	34 nm; spherical and irregular shape	Flavanoids and terpenoids	Ahmed et al. [88]
*Adathoda vasica*	Leaf	10–50 nm; spherical	Alkaloids compounds	Latha et al. [89]
*Amaranthus gangeticus*	Leaf	11–15 nm; globular and polycrystalline	Amino acids	Kolya et al. [90]
*Phlomis*	Leaf	25 nm; spherical	Glycosides such as flavonoids, iridoids, diterpenoids, triterpenoids and other phenolic compounds	Allafchian et al. [91]
*Syzygium alternifolium*	Fruit	4–48 nm; spherical	Phenols and primary amines of proteins	Yugandhar et al. [92]
*Afzelia quanzensis*	Bark	10–80 nm; spherical	Proteins	Moyo et al. [93]
*Allamanda cathartica*	Flower	39 nm; spherical	(E,E)-geranyl linalool, n-pentacosane, 1,8-cineole and n-tricosane	Karunakaran et al. [94]
*Carica papaya*	Peel	10–30 nm; spherical	Vitamins (C, K, E), amino acids, carbohydrates, β-carotene, lycopene and polyphenols	Kokila et al. [95]
*Vitis vinifera*	Leaf	200 nm; spherical	Hydroxyl groups and phenolic compounds mainly myricetin, ellagic acid, kaempferol and gallic acid	El-Sherbiny et al. [96]
*Solanum indicum*	Leaf	10–50 nm; spherical	Phenolic compounds	Sengottaiyan et al. [97]
*Tectona grandis*	Leaf	26–28 nm; spherical	Phenols	Devadiga et al. [98]
*Soymida febrifuga*	Leaf	10–20 nm; spherical	Phenolic groups, amino acids, aliphatic and aromatic amines, amide-I and amide-II	Sowmyyan and Lakshmi [99]
*Cardiospermum halicacabum*	Leaf	SEM-less than 100 nm; spherical	Polyphenols and phenol	Sundararajan et al. [100]
*Ammannia baccifera*		105–125 nm; spherical	Polyphenols, flavonoids and proteins	Jadhav et al. [101]
*Diospyros paniculata*	Root	17 nm (avg); spherical	Phenolics and proteins	Rao et al. [102]
*Simarouba glauca*	Leaf	33–50 nm; spherical	Amino groups and hydroxyl groups	Kanchana and Zantye [103]
*Origanum majorana* and *Citrus sinensis*	Leaf	40–70 nm; feather and 26–60 nm; spherical, cubical respectively	Proteins and phenolic compounds	Singh et al. [104]
*Salmalia malabarica*	Gum	7 ± 2 nm; spherical	Carbonyl and hydroxyl group	Krishna et al. [105]
*Psidium guajava*	Leaf	10–90 nm; spherical	Leucocyanidin, flavonoids, tannins, saponins, carotenes, vitamin C, B6 and carbohydrates	Bose and Chatterjee [106]
*Allium cepa*	Bulb	–	–	Balamanikandan et al. [107]
*Justicia glauca*	Leaf	10–20 nm; spherical	Phenolic compounds	Awad et al. [108]
*Skimmia laureola*	Leaf	Irregular, spherical, hexagonal	Tritepenoids, skimmidiol and coumarins	Ahmed et al. [109]
*Andrographis echioides*	Leaf	~ 68.06 nm; cubic	Carbohydrates, tannins, saponins, flavonoids, alkaloids, quinones, glycosides, triterpenoids, phenols, steroids, phytosteroids and anthraquinones	Elangovan et al. [110]
*Putranjiva roxburghii*	Leaf	5.74 nm; spherical	Amino groups	Ali et al. [111]
*Ixora coccinea*	Flower	5–10 nm; spherical	Alkaloids, tannins, glycosides, flavonoids, saponins, terpenes and carbohydrates	Nalvolthula et al. [112]
*Emblica officinalis*	Fruit	10–70 nm; spherical	Alkaloids, phenolic compounds, amino acids and tannins	Ramesh et al. [113]
*Hibiscus rosa*-*sinensis*	Petals	~ 18.79 nm; spherical	Proteins	Nayak et al. [114]
*Bauhinia variegata*	Leaf	32 nm; spherical, triangular, truncated triangles, decahedral	Reducing sugar, saponins, anthraquinone, alkaloids and terpenoids	Govindarajan et al. [115]
*Pteridium aquilinum*	Leaf	SEM-35–65 nm; spherical	Phenols, alkaloids, tannins, flavonoids, proteins, carbohydrates, saponins. glycosides, steroids and triterpenoids	Panneerselvam et al. [116]
*Aristolochia indica*	Leaf	30–55 nm; spherical and cubical	Phenols	Murugan et al. [117]
*Cassia roxburghii*	Leaf	~ 32 nm; spherical, triangular, truncated triangles, decahedral	–	Muthukumaran et al. [118]
*Anisomeles indica*	Leaf	TEM-18–35 nm; SEM-50–100 nm; spherical	Alcohols, phenols and carboxylic group	Govindarajan et al. [119]
*Hybanthus enneaspermus*	Plant	16–26 nm; spherical, hexagonal, triangular	Proteins	Suman et al. [120]
*Amaranthus dubius*	Leaf, stem, root	Stem: 30–35 nm; Root: 18–21 nm; Leaf: 18–21 nm	Polyphenol compounds and aldehydes	Sigamioney et al. [121]
*Ziziphus jujuba*	Fruit	25.75 nm; spherical	Alcohols and phenols	Sreekanth et al. [122]
*Chrysophyllum oliviforme*	Leaf	25 nm; flower	Flavonoids, saponins, catechic tannins, traces of anthraquinones, reducing sugars and phenolic compounds	Varghese et al. [123]
*Plumeria alba*	Flowers	~ 36.19 nm; spherical	Amino, carboxylic and sulfhydryls	Mata et al. [124]
*Impatiens balsamina*	Flowers	5–40 nm; spherical	Alkaloids, tannins, glycosides, flavonoids, saponins, terpenes and carbohydrates	Nalavothula et al. [125]
*Fraxinus excelsior*	Leaf	25–40 nm; spherical and polydisperse	Flavonoids, alkaloids, glycosides, terpenoids, phenolic compounds, amino acid residues and peptides of proteins	Parveen et al. [126]
*Pongamia pinnata*	Leaf	AFM-15–35 nm; spherical	Alkaloids, glycosides, flavonoids, saponins, carbohydrates, tannins, phenolic compounds and fat	Priya et al. [127]
*Pongamia pinnata*	Seed	5–30 nm; spherical	Pongaflavanol, tunicatachalcone, pongamol, galactoside and glybanchalcone	Beg et al. [128]
*Areca catechu*	Nut	18.2 and 24.3 nm; spherical	Polyphenols	Rajan et al. [129]
*Ficus talboti*	Leaf	9–12 nm; spherical	Flavonoids, alkaloids, saponins, phenolic compounds, tannins, phytosterol and glycosides	Arunachalam et al. [130]
*Sida cordifolia*	Leaf	10–30 nm; spherical, prism	Alkaloids, quinazolines, cryptoleptins, phytosterols, flavonoids and saponins	Srinithya et al. [131]
*Clerodendrum phlomidis*	Leaf	TEM 10–15 nm; SEM 23–42 nm; spherical	Phenolics, flavonoids, terpenoids and steroids	Sriranjani et al. [132]
*Theobroma cacao*	Pod husk	4–32 nm; face-centered cubic	Proteins and phenolic compounds	Lateef et al. [133]
*Ficus carica*	Fruit	20–80 nm (thermal approach), 10–30 nm (ultra sonication approach); spherical	–	Kumar et al. [134]
*Parkia speciosa* Hassk	Pod	20–50 nm; predominantly spherical	–	Fatimah [135]
*Boerhaavia diffusa*	Whole plants	25 nm; spherical		Vijay Kumar et al. [136]
*Pelargonium endlicherianum*	Roots	Different size; spherical	Gallic acid, apocynin and quercetin	Karatoprak et al. [137]
*Artocarpus heterophyllus*	Seeds	10.78 nm; irregular	Lectin—a single major protein	Jagtap and Bapat [138]
*Ceropegia thwaitesii*	Leaf	100 nm; spherical	Triterpenoids; and methoxy groups of protein	Muthukrishnan et al. [139]
*Alternanthera sessilis*	Leaf	30 nm; various shape	Alkaloid, tannins, ascorbic acid, carbohydrates and proteins	Niraimathi et al. [140]
*Dryopteris crassirhizoma*	Rhizome	5–60 nm; almost spherical	Alcohol, amines, alkanes, carboxylic acid and or ester	Lee et al. [141]
*Leptadenia reticulate*	Leaf	50–70 nm; crystalline, face centered and spherical	Phenolics, terpenoids, polysaccharides and flavones	Swamy et al. [142]
*Ipomoea batatas*	Root	TEM 30–120 nm; AFM 50–200 nm; polygonal	Glycoalkaloids, mucin, dioscin, choline, polyphenols and anthocyanins	Wang et al. [143]
*Sambucus nigra*	Fruit	26 nm; spherical	Polyphenols	Moldovan et al. [144]
*Millettia pinnata*	Flower	16–38 nm; spherical	Multi-functional aromatic gropus	Rajakumar et al. [145]
*Coptis chinensis*	Plant extract	15 nm; spherical	Polyphenols	Ahmad et al. [146]
*Lycium barbarum*	Fruit	3–15 nm; spherical	Tannias, flavanoids, ascorbic acid and alkaloids	Dong et al. [147]
*Embelia ribes*	Seed	20–30 nm; crystalline, uniform and spherical	Alkaloids, quinones, proteins, reducing sugars and saponins	Dhayalan et al. [148]
*Zizyphus xylopyrus*	Bark	60–70 nm; spherical	Reducing agents	Maria et al. [149]

**Table 2:** Bio-surfactants and or stabilizing agents used during synthesis of silver nanoparticles from various bacterial stains

Bacteria	Size and shape	Biosurfactants and or stabilizing agent	Key references
*Pseudomonas aeruginosa* BS-161R	15.1 ± 5.8 nm; spherical	Rhamnolipids	Kumar et al. [150]
*Brevibacterium casei* MSA19	–	Biosurfactant	Kiran et al. [151]
*Bacillus cereus* NK1	50–80 nm; spherical	URAK (a fibrinolytic enzyme)	Deepak et al. [152]
*Gluconacetobacter xylinum*	5–40 nm	Cellulose	Liu et al. [153]
*Streptomyces coelicolor*	28–50 nm; irregular	Actinorhodin pigment	Manikprabhu and Lingappa [154]
*Bacillus subtilis* MSBN 17	60; spherical	Bioflocculant	Sathiyanarayanan et al. [155]
*Salmonella typhimurium*	3–11 nm	Flagellin	Gopinathan et al. [156]
*Bacillus athrophaeus*	5–30 nm; polydispersed	Spores	Hosseini-Abari et al. [157]
*Lactobacillus rhamnosus* GG ATCC 53103	2–15 nm; spherical, triangular, rod-shaped and hexagonal	Exopolysaccharide	Kanmani and Lim [158]
*Nostoc commune*	15–54 nm; spherical	Extracellular polysaccharide/matrix	Morsy et al. [159]
*Pseudomonas aeruginosa*	1.13 nm; spherical	Biosurfactant	Farias et al. [160]
*Ochrobactrum rhizosphaerae*	10 nm; spherical	Glycolipoprotein	Gahlawat et al. [161]
*Gordonia amicalis* HS-11	5–25 nm; spherical	Glycolipid	Sowani et al. [162]
*Bacillus subtilis*	–	Surfactin	Mendrek et al. [163]

**Fig. 1:**
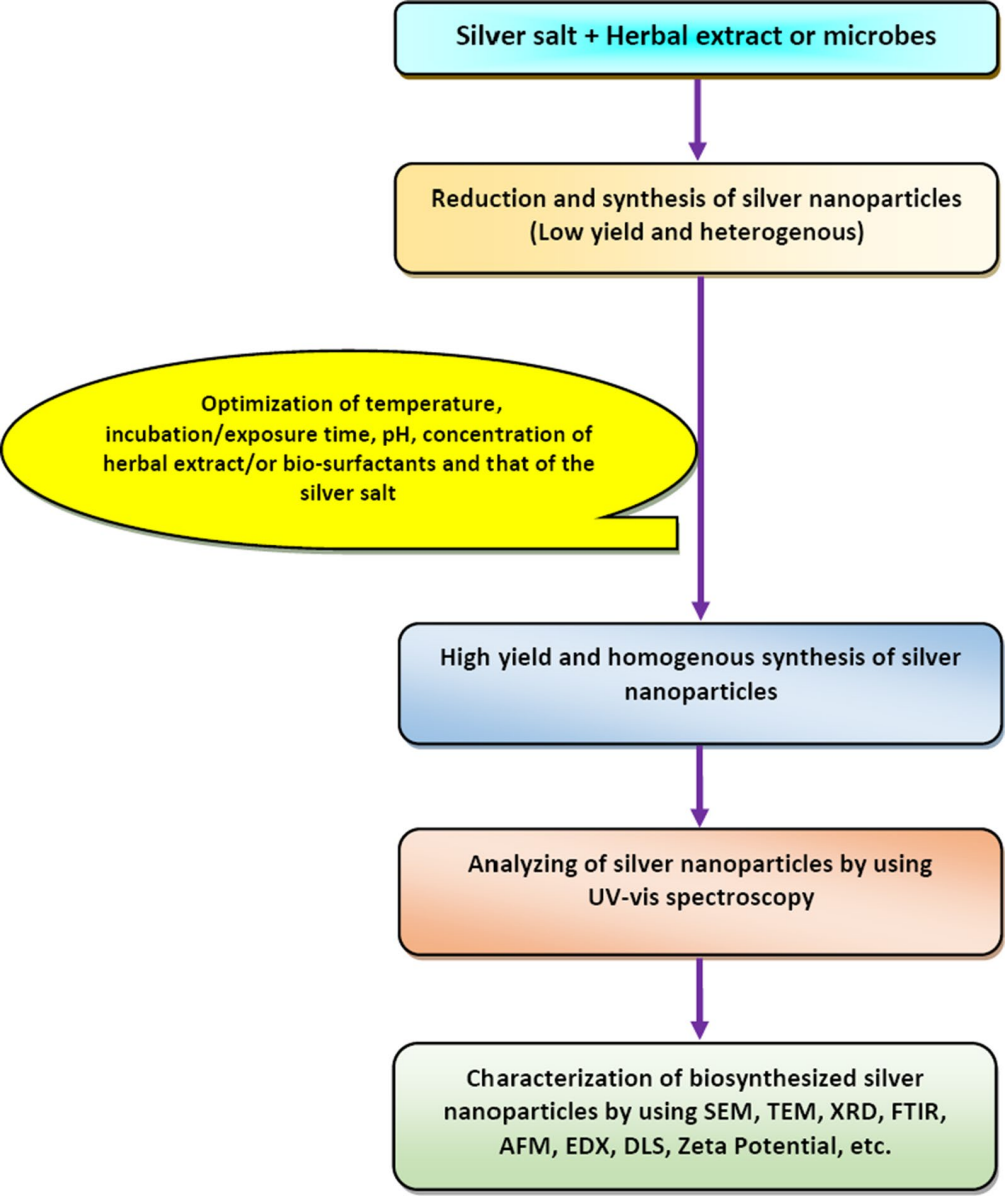
Biosynthesis of silver nanoparticles and their optimization techniques

Extracellular synthesis of Ag NPs comprises of the trapping of metal ions on the outer surface of the cells and reducing them in the presence of enzymes or biomolecules, while intracellular synthesis occurs inside the microbial cells. It has been suggested that the extracellular synthesis of nanoparticles is cheap, favors large-scale production and requires simpler downstream processing. Thus, the extracellular method for the synthesis of nanoparticles is preferable [164] in comparison to the intracellular method. Ganesh Babu and Gunasekaran [165] and Kalimuthu et al. [166] have demonstrated that the intracellular synthesis requires additional steps for instance, ultrasound treatment or reactions with suitable detergents to release the synthesized silver nanoparticles. Further, the rate of biosynthesis of Ag NPs and their stability is a significant part in industrial production. Therefore, a proper monitoring of reaction conditions is also important (Fig. 1).

### From bacteria

In recent years, the potential of biosynthesis of Ag NPs using bacteria has been realized [15, 153, 156–159]. For instance, *Pseudomonas stutzeri* AG259—isolated from silver mine was used to produce Ag NPs inside the cells [167]. In addition, several bacterial strains (gram negative as well as gram positive) namely *A. calcoaceticus, B. amyloliquefaciens, B. flexus, B. megaterium* and *S. aureus* have been used for both extra- and intracellular biosynthesis of Ag NPs [168–174]. These Ag NPs are spherical, disk, cuboidal, hexagonal and triangular in shape. They have been fabricated using culture supernatant, aqueous cell-free extract or cells (Table 3). Saifuddin et al. [14] have demonstrated an extracellular biosynthesis of Ag NPs (∼ 5–50 nm) using a combination of culture supernatant of *B. subtilis* and microwave irradiation in water. Shahverdi et al. [15] have reported rapid biosynthesis of Ag NPs (within 5 min) using the culture supernatants of *K. pneumonia*, *E. coli* and *Enterobacter cloacae*. Saravanan et al. [172] have also reported an extracellular synthesis of Ag NPs using *B. megaterium* cultured supernatant, within minutes in presence of aqueous solutions of Ag^+^ ions.

**Table 3:** Bacteria-mediated synthesis of silver nanoparticles

Bacteria	Size and shape	Location	Key references
*Acinetobacter calcoaceticus*	8–12 nm; spherical	Extracellular	Singh et al. [175]
*A. haemolyticus* MMC8	4–40 nm	Extracellular	Gaidhani et al. [176]
*Aeromonas* sp. SH10	6.4 nm	Extracellular and intracellular	Mouxing et al. [177] Wang et al. [178]
*Bordetella* sp.	63–90 nm	Extracellular	Thomas et al. [179]
*Enterobacter aerogenes*	25–35 nm; spherical	Extracellular	Karthik and Radha [180]
*Escherichia coli*	42.2–89.6 nm; spherical	Extracellular	Gurunathan et al. [181]
*Geobacter sulfurreducens*		Extracellular	Law et al. [182]
*Gluconobacter roseus*	10 nm	Extracellular	Krishnaraj and Berchmans [183]
*Idiomarina* sp.	25 nm	Intracellular	Seshadri et al. [184]
*Klebsiella pneumoniae*	15–37 nm; spherical	Extracellular	Duraisamy and Yang [185]
	5–32 nm	Extracellular	Shahverdi et al. [15]
*Morganella* sp.	10–40 nm; quasispherical	Extracellular	Parikh et al. [186]
*Proteus mirabilis*	10–20 nm; spherical	Extracellular and intracellular	Samadi et al. [187]
*Pseudomonas aeruginosa* SM1	6.3 ± 4.9 nm; spherical, disk-shaped	Extracellular	Srivastava and Constanti [188]
	8–24 nm; spherical	Extracellular	Kumar and Mamidyala [189]
	5–25 nm; quasispherical	Intracellular	Otaqsara [190]
*Rhodobacter sphaeroides*	Spherical 3–15	Extracellular	Bai et al. [191]
*Rhodopseudomonas palustris*	Spherical 5–20	Extracellular	Chun-Jing and Hong-Juan [192]
*Shewanella oneidensis* MR-1	2–16 nm; spherical (Ag2S)	Extracellular	Debabov et al. [193]
*Stenotrophomonas maltophilia*	93 nm; cuboidal	Extracellular	Oves et al. [194]
*Vibrio alginolyticus*	50–100 nm; Spherical	Extracellular and intracellular	Rajeshkumar et al. [195]
*Xanthomonas oryzae*	14.86 nm; spherical, triangular, rod-shaped	Extracellular	Narayanan and Sakthivel [196]
*Yersinia enterocolitica*	10–80 nm	Extracellular	Pourali et al. [197]
*Bacillus* sp.	5–15 nm	Extracellular and periplasmic space	Pugazhenthiran et al. [198]
*B. cereus*	4–5 nm; spherical	Intracellular	Ganesh Babu and Gunasekaran [165]
*B. flexus*	12 and 65 nm; spherical and triangular	Extracellular	Priyadarshini et al. [173]
*B. licheniformis* Dahb1	18.69–63.42 nm; spherical	Cell free extract	Shanthi et al. [199]
*B. safensis* LAU 13	5–30 nm; spherical	Extracellular	Lateef et al. [200]
*B. methylotrophicus* DC3	10–30 nm; spherical	–	Wang et al. [201]
*B. subtilis*	Triangular, hexagonal	Extracellular	Kannan et al. [202]
*B. subtilis* MTCC 3053	20–60 nm; polydispersed(AgCl)	–	Paulkumar et al. [203]
*B. thuringiensis*	43.52–142.97 nm	Extracellular	Banu et al. [204]
*Brevibacterium casei*	10–50 nm; spherical	Intracellular	Kalishwaralal et al. [205]
*Corynebacterium* SH09	10–15 nm	Extracellular	Zhang et al. [206]
*Enterococcus faecalis*	10–80 nm	Extracellular	Pourali et al. [197]
*Exiguobacterium* sp.	5–50 nm; spherical	Extracellular	Tamboli and Lee [207]
*Geobacillus stearothermophilus*	5–35 nm; spherical	Extracellular	Fayaz et al. [208]
*Lactobacillus mindensis*	2–20 nm; spherical (Ag_2_O)	Extracellular	Dhoondia and Chakraborty [209]
*Rhodococcus* sp.	10–15 nm; spherical	Extracellular	Otari et al. [210]
*Staphylococcus epidermidis*	10–80 nm	Extracellular	Pourali et al. [197]
*Thermoactinomyces* sp.	20–40 nm; spherical	Extracellular	Deepa et al. [211]
*Ureibacillus thermosphaericus*	10–100 nm; spherical	Extracellular	Juibari et al. [212]

Rapid synthesis of Ag NPs has been achieved by the interaction of a bacterial strain S-27, belonging to *Bacillus flexus* group and 1 mM AgNO_3_ in aqueous medium [173]. The colourless supernatant solution turned yellow and finally brown. Its UV–vis spectrum exhibited a sharp peak at 420 nm due to the surface plasmon resonance (SPR) of silver nanoparticles. Anisotropic nanoparticles of 12 and 65 nm size were stable in the dark for 5 months at room temperature although their slow degradation cannot be prevented. They were crystalline with a face centered cubic structure. These nanoparticles were found to be effective against multidrug resistant gram positive and gram negative bacteria. The colour intensity and rate of interaction depend on the concentration of the reacting components.

Das et al. [174] have reported extracellular biosynthesis of Ag NPs from the *Bacillus* strain (CS11). The interaction of 1 mM AgNO_3_ with the bacteria at room temperature yielded nanoparticles within 24 h which showed a peak at 450 nm in UV–vis spectrum. Their size from TEM analysis was found to range between 42 and 92 nm (Table 3).

### From fungi

Biosynthesis of Ag NPs from both pathogenic and nonpathogenic fungi has been investigated extensively [10, 164, 213–215] (Table 4). It has been reported that silver ions are reduced extracellularly in the presence of fungi to generate stable Ag NPs in water [214, 216].

**Table 4:** Fungus-mediated synthesis of silver nanoparticles

Fungus	Size and shape	Location	Key references
*Aspergillu flavus*	8.92 nm; spherical	Cell wall	Vigneshwaran et al. [217]
*A. fumigatus*	–	Extracellular	Bhainsa and D’Souza [218]
*A. terreus*	1–20 nm; spherical	Extracellular	Li et al. [219]
*Cladosporium cladosporioides*	10–100 nm	–	Balaji et al. [220]
*Coriolus versicolor*	25–75, 444–491 nm; spherical	Extracellular and intracellular	Sanghi and Verma [221]
*Fusarium oxysporum*	–	Extracellular	Ahmad et al. [222]
	20–50 nm; spherical	Extracellular	Durán et al. [164]
	5–50 nm	–	Senapati et al. [223]
*Humicola* sp.	5–25 nm; spherical	Extracellular	Syed et al. [224]
*Macrophomina phaseolina*	5–40 nm; spherical	Cell-free filtrate	Chowdhury et al. [225]
*Pediococcus pentosaceus*	–	Extracellular	Shahverdi et al. [15]
*Penicillium brevicompactum*	58.35 ± 17.88 nm	–	Shaligram et al. [226]
*P. fellutanum*	5–25 nm; spherical	Extracellular	Kathiresan et al. [215]
*P. nalgiovense* AJ12	25 ± 2.8 nm; spherical	Cell-free filtrate	Maliszewska et al. [227]
*Phaenerochaete chrysosporium*	5–200 nm; pyramidal	–	Vigneshwaran et al. [228]
*Phoma glomerata*	60–80 nm; spherical	–	Birla et al. [229]
*Pleurotus ostreatus*	< 40 nm; spherical	–	Al-Bahrani et al. [230]
*P. sajor*-*caju*	30.5 ± 4.0 nm; spherical	Extracellular	Vigneshwaran et al. [231]
*Trichoderma asperellum*	13–18 nm; nanocrystalline	Extracellular	Mukherjee et al. [232]
*T. reesei*	5–50 nm	Extracellular	Vahabi et al. [233]
*T. viride*	5–40 nm; spherical	Extracellular	Fayaz et al. [234]
*T. viride*	2–5 nm; spherical 40–65 nm; rectangular 50–100 nm; penta/hexagonal (Obtained at varying pH, reaction time and temperature of the reaction mixture)	Cell free extract	Kumari et al. [235]

Syed et al. [224] have also reported the extracellular synthesis of Ag NPs from thermophilic fungus *Humicola* sp. All manipulations were done in aqueous medium at room temperature. Mycelia were suspended in 100 mL of 1 mM AgNO_3_ solution in an Erlenmeyer flask at 50 °C and the mixture was left in a shaker for 96 h at pH 9 and monitored for any change in colour. The solution showed a change in colour from yellow to brown due to the formation of Ag NPs [222]. It is a simple process for the extracellular synthesis of Ag NPs from *Humicola* sp. TEM micrograph showed nicely dispersed nanoparticles mainly of spherical shape ranging between 5 and 25 nm. They are crystalline with a face centered cubic structure [236]. IR spectrum of Ag NPs in the suspension showed peaks at 1644 and 1523 cm^−1^ assigned to amide I and amide II bands of protein corresponding to –C=O and N–H stretches. Owaid et al. [237] have reported the biosynthesis of Ag NPs from yellow exotic oysters mushroom, *Pleurotus cornucopiae* var. *citrinopileatus*. The dried basidiocarps were powdered, boiled in water and the supernatant was freeze dried. Different concentrations of hot water extract of this lyophilized powder were mixed with 1 mM AgNO_3_ at 25 °C and incubated for 24, 48 and 72 h. Change in colour from yellow to yellowish brown exhibited an absorption peak at 420 and 450 nm in UV–vis region which is the characteristic of spherical silver nanoparticles. The width of the absorption peak suggests the polydispersed nature of nanoparticles [221]. IR spectrum of Ag NPs exhibited absorption peaks at 3304, 2200, 2066, 1969, 1636, 1261, 1094 and 611 cm^−1^ for different groups. Although, authors have indicated the presence of polysaccharide and protein in the mushroom they have ignored their stretching frequencies in the IR spectrum. However, the peak at 3304 has been assigned to υ (OH) of carboxylic acid and those at 2200 and 1969 cm^−1^ have been attributed to unsaturated aldehydes. The other peaks below 1500 cm^−1^ are due to unsaturated alkaloids. The field emission scanning electron and high-resolution transmission electron micrograph suggested that the Ag NPs are spherical with average size ranging between 20 and 30 nm.

Very recently, Al-Bahrani et al. [230] reported biogenic synthesis of Ag NPs from tree oyster mushroom *Pleurotus ostreatus*. Dried aqueous extract of mushroom (1–6 mg/mL) and 1 mM AgNO_3_ were mixed and incubated in the dark for 6–40 h. The colour change from pale yellow to dark brownish yellow indicated the formation of silver nanoparticles. The UV–vis spectrum showed a sharp and broad absorption band at 420 nm. They are polydispersed nanoparticles of 10–40 nm with an average size of 28 nm. Several fungi namely, *Aspergillus flavus*, *A. fumigates*, *Fusarium oxysporum, Fusarium acuminatum*, *F. culmorum*, *F. solani*, *Metarhizium anisopliae, Phoma glomerate, Phytophthora infestans, Trichoderma viride, Verticillium* sp. have been used for both extra- and intracellular biosynthesis of Ag NPs [10, 164, 216–219, 222]. These nanoparticles are of various sizes and shapes (Table 4).

### From plants

Plant related parts such as leaves, stems, roots, shoots, flowers, barks, seeds and their metabolites have been successfully used for the efficient biosynthesis [1, 238] of nanoparticles (Fig. 1). Very recently, Beg et al. [128] have reported green synthesis of Ag NPs from seed extract of *Pongamia pinnata*. The formation of nanoparticles was confirmed by an absorption max at 439 nm. The well dispersed nanoparticles with an average size of 16.4 nm had zeta potential equal to − 23.7 mV which supports dispersion and stability. Interaction of Ag NPs with human serum albumin was investigated and showed negligible change in α helics. In a very recent publication Karatoprak et al. [137] have reported green synthesis of Ag NPs from the medicinal plant extract *Pelargonium endlicherianum*. The plant containing gallic acid, apocyanin and quercetin act as reducing agents to produce silver nanoparticles. Phytomediated synthesis of spherical Ag NPs from *Sambucus nigra* fruit extract has been reported by Moldovan et al. [144]. XRD analysis showed them to be crystalline. The in vivo antioxidant activity was investigated against Wistar rats which showed promising activity. It suggests that functionalization of Ag NPs with natural phytochemicals may protect the cell proteins from ROS production. Ag NPs have also been synthesized from aqueous leaf extract of *Artocapus altilis*. They were moderately antimicrobial and antioxidant. *Thalictrum foliolosum* root extract mediated Ag NPs synthesis has been confirmed on the basis of the appearance of a sharp peak at 420 nm in UV–vis region of the spectrum [239]. The monodispersed spherical nanoparticle of 15–30 nm had face centered cubic geometry. Shape and size dependent controlled synthesis of Ag NPs from *Aloe vera* plant extract and their antimicrobial efficiency has been reported by Logaranjan et al. [35]. The UV–vis peak at 420 nm confirmed the formation of silver nanoparticles. After microwave irradiation of the sample, Ag NPs of 5–50 nm with octahedral geometry was obtained. Nearly two to fourfold antibacterial activity of Ag NPs was observed compared to commonly available antibiotic drugs. Biosynthesis of Ag NPs from the aqueous extract of *Piper longum* fruit extract has been also achieved [240]. The nanoparticles were spherical in shape with an average particle size of 46 nm determined by SEM and dynamic light scattering (DLS) analyser. The polyphenols present in the extract are believed to act as a stabilizer of silver nanoparticles. The fruit extract and the stabilized nanoparticles showed antioxidant properties in vitro. The nanoparticles were found to be more potent against pathogenic bacteria than the flower extract of *P. longum*. Ag NPs have been fabricated from leaf extract of *Ceropegia thwaitesii* and formation was confirmed from absorption of SPR at 430 nm. The nanoparticles of nearly 100 nm diameter were crystalline in nature [139]. Plant extract of *Ocimum tenuiflorum*, *Solanum tricobatum*, *Syzygium cumini*, *Centella asiatica* and *Citrus sinensis* have been used to synthesize Ag NPs of different sizes in colloidal form [249]. The size of all nanoparticles was found to be 22–65 nm. They were all stable and well dispersed in solution. Niraimathi and co-workers [140] have reported biosynthesis of Ag NPs from aqueous extract of *Alternanthera sessilis* and showed that the extract contains alkaloids, tannins, ascorbic acid, carbohydrates and proteins which serve as reducing as well as capping agents. Biomolecules in the extract also acted as stabilizers for silver nanoparticles. Ag NPs from seed powder extract of *Artocarpus heterophyllus* have been synthesized [138]. The morphology and crystalline phase of the nanoparticles were determined by SEM, TEM and SAED, EDAX and IR spectroscopy. They were found to be irregular in shape. The extract was found to contain amino acids, amides etc. which acted as reducing agents for AgNO_3_ to produce silver nanoparticles. The quantity of phenols, anthocyanins and benzoic acid were determined in the berry juices and were responsible for the transformation of silver ions to Ag NPs [241]. UV–vis spectra displayed an absorbance peak at 486 nm for lingonberry and 520 nm for cranberry containing silver nanoparticles. Since the two absorption peaks are different they cannot be assigned only to Ag NPs but also partly to different quantities of the reducing chemicals present in the juices. However, the spectra indicated the presence of polydispersed silver nanoparticles. Puiso et al. [241] have proposed that due to irradiation of water by UV rays, strong oxidants and reductants as photolysis products are formed. They reduce silver ions to Ag NPs or silver oxide. The photolysis products may produce oxidant and reductant but it depends upon the quantum of radiation and exposure time which may not be enough to produce a sufficient quantity of redox chemicals to reduce Ag^+^ to Ag NPs or Ag_2_O. This hypothesis is conceptually incorrect because Ag_2_O cannot be formed as it requires a very strong oxidizing agent. On the other hand, AgNO_3_ itself is slowly reduced in water, but in the presence of reducing agents the reaction proceeds at a rapid rate. The SPR is dependent on the size, shape and agglomeration of Ag NPs which is reflected from the UV–vis spectra [242]. Mock et al. [243] have found different scattered colors in hyperspectral microscopic images which are mainly due to the different shape and size of silver nanoparticle in the colloidal solution. The blue, green, yellow and red colors have been attributed to spherical, pentagonal, round-triangle and triangle shapes, respectively.

**Fig. 2:**
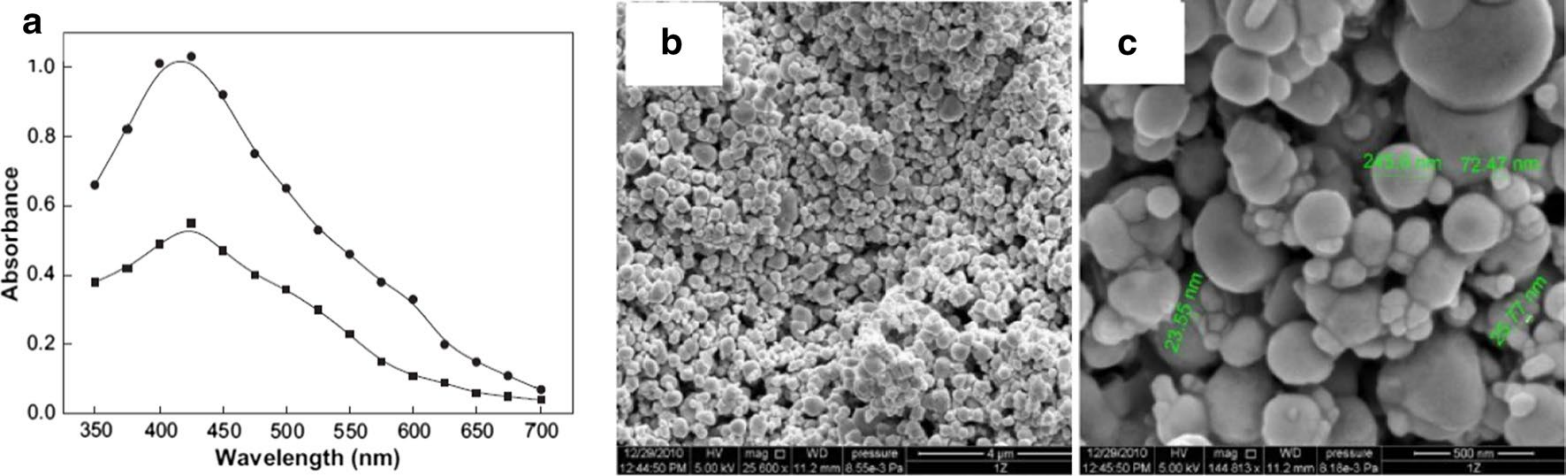
**a** UV–visible spectra of yellow color silver solution. **b** and **c** SEM images of the self-assembled silver nanoparticle mirror like illumination on the walls of the glass. Reaction conditions: [Ag^+^] = 20.0 × 10^−4^ mol dm^−3^; [oxalic acid] = 4.0 × 10^−4^ mol dm^−3^; [CTAB] = 10.0 × 10^−4^ mol dm^−3^; temperature = 30 °C [12]

**Scheme 1:**
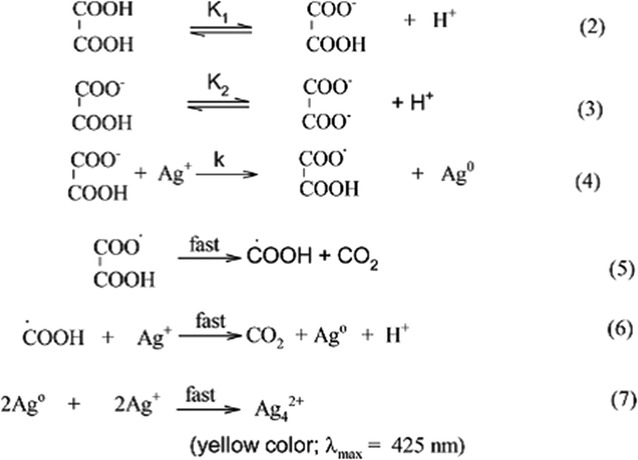
Reduction of Ag^+^ ions by oxalic acid [12]

Zaheer and Rafiuddin [12] have reported the synthesis of Ag NPs using oxalic acid as reducing agent and mistook it as green synthesis. Formation of nanoparticles was confirmed by a change in color of the solution which showed an absorption peak at 425 nm (Fig. 2a) in the UV–visible region. It was also noted that a scattered silver film was formed on the wall of the container that shines and reflects light (Fig. 2b) which is the characteristic of monodispersed spherical Ag NPs [244, 245]. Since the size of nanoparticles varies between 7 and 19 nm the silver film is not uniform. It is different from regular silver mirror due to irregular shape and size of nanoparticles (Fig. 2c). Actually, very small size nanoparticles can be obtained when AgNO_3_ is exposed to a reducing agent for a longer duration of time [246]. The kinetics and mechanism proposed for the formation of Ag NPs by oxalic acid is not convincing [12] because oxalic acid in no case can produce CO_2_ unless it reacts with any carbonate salt or heated at a very high temperature. The authors [12] have proposed following reactions to prove that the colour of Ag NPs in solution is due to Ag_4_^2+^ formation that absorbs at 425 nm (Scheme 1). The formation of Ag_4_^2+^ is highly improbable even if the above reaction is kinetically very fast. Also, the stabilization of Ag_4_^2+^ is questionable (Scheme 1). This hypothesis of Ag_4_^2+^ formation is beyond imagination and does not carry any experimental evidence in its support. Absorbance of Ag NPs in solution varies between 400 and 445 nm depending on the nature of reducing agent used for their fabrication. The SPR band in UV–vis spectrum is due to electron oscillation around the surface of nanoparticles. The reduction process is instantaneous and no further spectral change occurs after 60 min. Indicating the completion of redox process. Ag NPs are circular, triangular, hexagonal and polydispersed at 70 °C. The EDAX and XRD spectra support each other.

**Fig. 3:**
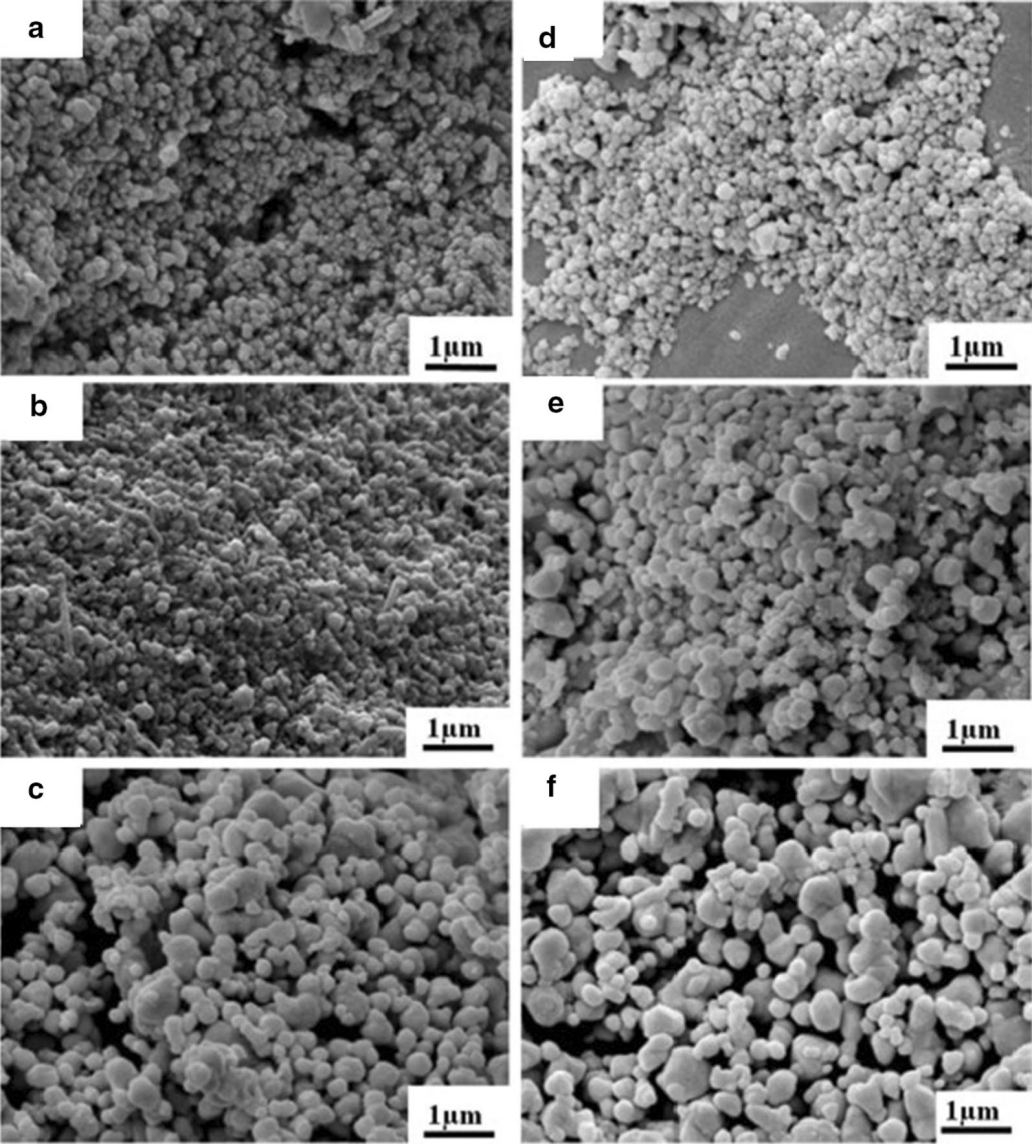
SEM images of silver nanoparticles were obtained at **a** 100 °C for 6 h, **b** 150 °C for 6 h, **c** 200 °C for 6 h, **d** 100 °C for 12 h, **e** 150 °C for 12 h and **f** 200 °C for 12 h [36]

Synthesis of Ag NPs from aqueous extract of *Cleistanthus collinus* and their characterization by UV–vis, FTIR, SEM, TEM and XRD has been reported by Kanipandian et al. [247]. The crystalline Ag NPs of 20–40 nm showed significant free radical scavenging capacity. Tippayawat et al. [27] have reported a green and facile synthesis of Ag NPs from *Aloe vera* plant extract. They were characterized by UV–vis, SEM, TEM and XRD. Fabrication of Ag NPs was confirmed on the basis of the appearance of a sharp peak at 420 nm in UV–vis region of the spectrum. In addition, they have reported that the reaction time and temperature markedly influence the fabrication of silver nanostructures. Ag NPs were spherical in shape and particle size ranged from 70.70 ± 22 to 192.02 ± 53 nm. Their size changes with time and temperature of the reaction mixture used during fabrication (Fig. 3).

Green synthesis of Ag NPs from *Boerhaavia diffusa* plant extract has been reported by Vijay Kumar et al. [136] where the extract acted as both the reducing as well as capping agent. The colloidal solution of Ag NPs showed an absorption maximum at 418 nm in the UV–vis spectrum. The XRD and TEM analyses revealed a face centered cubic structure with an average particle size of 25 nm. Ag NPs of 5–60 nm have been synthesized from *Dryopteris crassirhizoma* rhizome extract in presence of sunlight/LED in 30 min [235]. XRD studies showed face centered cubic structure of silver nanoparticles.

Green synthesis of Ag NPs using 1 mM aqueous AgNO_3_ and the leaf extract of *Musa balbisiana* (banana), *Azadirachta indica* (neem) and *Ocimum tenuiflorum* (black tulsi) has been done [248]. They were characterized by UV–vis, SEM, TEM, DLS, EDS and FTIR spectroscopy. They were found to accelerate the germination rate of *Vigna radiata* (Moong Bean) and *Cicer arietinum* (Chickpea). It is therefore, believed that Ag NPs are not toxic to such crops at germination level. Stable and capped Ag NPs from aqueous fruit extract of *Syzygium alternifolium* of 5–68 nm have been synthesized [92]. Nearly 12.7% of silver was detected from EDAX. The polydispersed spherical nanoparticles were capped and stabilized by the phenols and proteins present in the fruit extract. Biosynthesis of Ag NPs from methanolic leaf extract of *Leptadenia reticulate* has been done [142]. They were crystalline, face centred and spherical particles of 50–70 nm. They exhibited antibacterial activity and radical scavenging activity. Purple sweet potato (*Ipomoea batatas* L.) root extract has been exploited to synthesize Ag NPs [143]. Organic components in the extract acted both as reducing and capping agents. Ag NPs have shown remarkable antibacterial activity against four clinical and four aquatic pathogens. Sweet potato root extract is known to contain glycoalkaloids, mucin, dioscin, choline, polyphenols and anthocyanins which function as antioxidant, free radical scavenger, antibacterial agent and reducing agents. In presence of Ag NPs these functions are further enhanced.

## Cytotoxicity of silver nanoparticles

Cytotoxicity of nanomaterials depends on their size, shape, coating/capping agent and the type of pathogens against which their toxicity is investigated. Nanoparticles synthesized from green method are generally more toxic than those obtained from the non-green method. Some pathogens are more prone to nanomaterials, especially Ag NPs than others due to the presence of both the Ag ions released and Ag NPs. They slowly envelop the microbes and enter into the cell inhibiting their vital functions. It is clear that the fabrication and application of nanoparticles has resulted in public awareness of their toxicity and impact on the environment [249, 250]. Nanoparticles are relatively more toxic than bulk materials. They are toxic at cellular, subcellular and biomolecular levels [251]. Oxidative stress and severe lipid peroxidation have been noticed in fish brain tissue on exposure to nanomaterials [252]. The cytotoxicity by Ag NPs is believed to be produced through reactive oxygen species (ROS) as a consequence of which a reduction in glutathione level and an increase in ROS level occur. From in vitro studies on animal tissue and cultured cells, Kim and Ryu [253] have observed an increase in oxidative stress, apoptosis and genotoxicity when exposed to silver nanoparticles. Since such studies have been made with varying sizes of Ag NPs and coatings under different conditions a direct correlation cannot be made. Hackenberg and coworkers [254] reported reduced viability at a dose of 10 µg/mL of Ag NPs of over 50 nm size in human mesenchymal cells whereas some people reported no toxicity [255] even at a higher dose (100 µg/mL). Besides, stability and aging of the sample are also important factors as an increase in toxicity has been reported by aged Ag NPs stored in water for 6 months which is related to the release of silver ions [256]. It seems that the toxicity is a cumulative effect of Ag NPs and silver ions. Some workers have shown that the toxicity of Ag NPs is due to released Ag ions [257] while others have attributed the toxicity to Ag NPs [258].

Vijay Kumar et al. [136] obtained Ag NPs from *B. diffusa* plant extract and tested them against three fish bacterial pathogens. It was found that Ag NPs were most effective against *Flavobacterium branchiophilum*. Ag NPs fabricated from *P. longum* fruit extract exhibited cytotoxic effect against MCF-7 breast cancer cell lines with an IC_50_ of 67 μg/mL/24 h [240]. They also exhibited antioxidant and antimicrobial effects. Ag NPs were produced by using *P. endlicherianum* plant extract; and have shown that the inhibitory activity was increased against gram positive and gram negative bacteria when they were exposed to Ag NPs at a very low dose of 7.81 to 6.25 ppm [137]. Latha et al. [89] have fabricated Ag NPs from leaf extract of *Adathoda vasica* and studied their antimicrobial activity against *Vibrio parahaemolyticus* in agar medium. The nanoparticles were found to be significantly active against *V. parahaemolyticus* but were nontoxic to *Artemia* nauplii. *V. parahaemolyticus* is a prevalent sea food borne enteropathogen which is closely associated with mortality in Siberian tooth carps, milk fish [259], abalone [260] and shrimps [251]. *Vibrio* infection in cultured fish and shrimps causes large scale mortality. Quite often, the whole population perishes. The use of antibiotic has made them resistant. Under such conditions, Ag NPs have appeared as an effective remedy which saves shrimps from perishing. Ag NPs from seed powder extract of *A. heterophyllus* have also exhibited antibacterial activity against gram positive and gram negative bacteria [138].

Ag NPs fabricated from leaf extract of *C. thwaitesii* have shown antibacterial efficacy against *Salmonella typhi*, *Shigella flexneri* and *Klbsiella pneumoniae* indicating them to be significant. Niraimathi and co-workers [140] have also fabricated Ag NPs from aqueous extract of *A. sessilis* and showed significant antibacterial and antioxidant activities. Ag NPs from *Ocimum tenuiflorum*, *Solanum tricobatum*, *Syzygium cumini*, *Centella asiatica* and *Citrus sinensis* have also shown antibacterial activity against *S. aureus*, *P. aeruginosa*, *E. coli* and *K. pneumoniae*. The highest activity of nanoparticles was observed against *S. aureus* and *E. coli* [261]. Antimicrobial activity of colloidal Ag NPs was found to be higher than the plant extract alone. Lee et al. [141] synthesized Ag NPs from *Dryopteris crassirhizoma* and found them to be highly effective against *B. cereus* and *P. aeruginosa*. Similarly, Ag NPs obtained from leaf extract of banana, neem and black tulsi were also active against *E. coli* and *Bacillus* sp. [248]. Hazarika et al. [239] have performed antimicrobial screening of Ag NPs obtained from *T. foliolosum* root extract against six bacteria and three fungi which showed morphological changes in the bacterial cells. Fabricated of Ag NPs from *Millettia pinnata* flower extract and their characterization together with anti-cholinesterase, antibacterial and cytotoxic activities have been reported by Rajakumar et al. [145]. Spherical shaped Ag NPs ranging from 16 to 38 nm exhibited excellent inhibitory efficacy against acetyl cholinesterase and butyl cholinesterase. They also exhibited cytotoxic effects against brine shrimp.

**Fig. 4:**
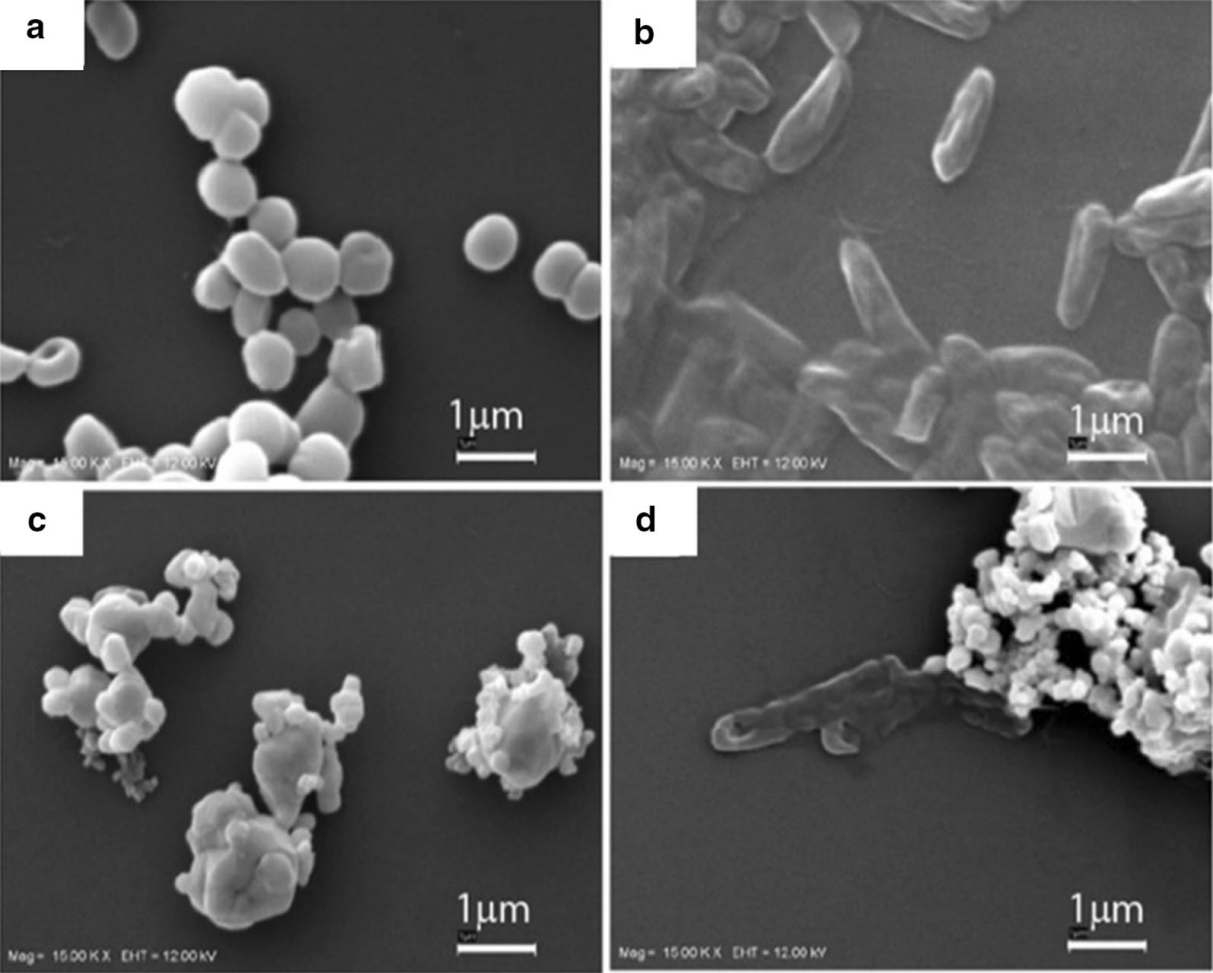
SEM images of the bacterial strains. **a** *Staphylococcus epidermidis*, Gram-positive, **b** *Pseudomonas aeruginosa*, Gram-negative, **c** *S. epidermidis* treated with 100-6 h silver nanoparticles (0.04 mg/mL), **d** *P. aeruginosa* treated with 100–6 h silver nanoparticles (0.04 mg/mL) [36]

Ag NPs obtained from *S. alternifolium* have also exhibited high toxicity towards bacterial and fungal isolates [92]. Ag NPs fabricated from *L. reticulate* [142] were found to be toxic to HCT15 cancer cell line. Kanipandian et al. [247] have reported that Ag NPs obtained from *C. collinus* aqueous extract exhibit dose dependent effects against human lung cancer cell (A549) and normal cell (HBL-100). The IC_50_ for cancer cells was very low (30 µg/mL) but since Ag NPs synthesized from *C. collinus* were toxic to normal cells they cannot be used in vivo. However, if the plant extract contains some antioxidants, the whole mixture may exhibit this property but the nanoparticles alone are incapable to do so. Ag NPs from *Aloe vera* plant extract have shown varying degrees of antibactericidal effects [36]. Ag NPs obtained at 100 °C for 6 h and 200 °C for 12 h (varying temperature and reaction time) exhibited change in bacterial cell membrane when contacted with the nanoparticles (Fig. 4). They were more effective for gram negative bacteria (*P. aeruginosa*, ATCC27803). In addition, they have also shown minimal cytotoxicity to human peripheral blood mononuclear cells.

The particle size, agglomeration and sedimentation are related to the cytotoxicity of silver nanoparticles. It has been demonstrated from Alamar Blue (AB) and Lactate dehydrogenase test (LDH) that Ag NPs of 10 nm coated with citrate and PVP separately, are toxic to human lung cells [262] when exposed for 24 h. AB test is a measure of cell proliferation and mitochondrial activity. However, the LDH measures the cytotoxicity of Ag NPs in terms of membrane damage from the cytoplasm. Both the citrate and PVP coated nanoparticles of 10 nm exhibited significant toxicity after 24 h at the highest dose of 50 µg/mL. Ag NPs of larger dimensions did not alter cell viability [263, 264]. Cytotoxicity is related to enzyme inhibition which is correlated to the release of Ag ions because they inhibit the catalytic activity of LDH.

It has been observed that Ag NPs damaged DNA but they did not increase ROS when cells were exposed to them for 24 h at a dose of 20 µg/mL [263]. Gliga et al. [262] have suggested that silver ions from AgCl are released in the biological fluid and complexed. The formation of AgCl is possible only if the fluid is contaminated with Cl^−^ ions, nevertheless it cannot ionize to Ag^+^ and Cl^−^ ions since AgCl is almost insoluble in aqueous medium [265]. The experiment with extracellularly released silver ions in cell medium did not exhibit toxicity, perhaps it would have reacted with Cl^−^ ions to yield insoluble AgCl.

Cytotoxicity is related to the size of Ag NPs irrespective of the coating agent. Carlson et al. [266] have shown an increase in ROS production for 15 nm hydrocarbon coated Ag NPs relative to 55 nm. It has been reported by Liu et al. [267] that 5 nm Ag-nanoparticles were more toxic than 20 and 50 nm nanoparticles to four cell lines, namely, A549, HePG2, MCF-7 and SGC-7901. Wang et al. [268] have also reported that smaller nanoparticles (10–20 nm) induce greater cytotoxicity than the larger ones (110 nm), and citrate coated 20 nm Ag NPs produced acute neutrophilic inflammation in the lungs of mice compared to those with larger ones. The cell viability and DNA damage may be explained by ROS generation [269] which may be contradictory to findings by others in in vitro studies [253].

It is hypothesized that irreparable DNA damage is due to the interaction of Ag NPs with repair pathways. Since this work has been done in vitro, the DNA once damaged may not have the ability to repair. However, in living systems the cells have the ability to undergo repair and multiply but such experiments have seldom been done. It is however, unanimously agreed that both Ag NPs and silver ions are present at the subcellular level. The transformation of Ag to Ag^+^ ions occurs due to their interaction with biomolecules in the cell membrane. The release of elemental silver is directly proportional to the size of nanoparticles in a non-linear fashion [270]. The size dependent toxicity is related to the intracellular release of silver ions. Although, agglomeration of nanoparticles reduces their release, the antibacterial effect was hindered under anaerobic condition, because in absence of oxygen, the oxidation process Ag → Ag^+^ ceases to continue. Ag NPs exhibited excellent activity against *Y. enterocolitica*, *P. vulgaris*, *E. coli*, *S. aureus* and *S. faecalis*. Since the nanoparticles are smaller than the bacterial cell they may stick to their cell walls disallowing permeation of essential nutrients leading to the death of microorganisms [236]. Smaller size is related to greater surface area of nanoparticles and their agglomeration around the cell wall inhibits the cell division of microbes.

Besides their application in diverse areas, Ag NPs are extensively used as antioxidant and antimicrobial agents regardless of the process of their synthesis [271, 272]. They are more toxic to microorganisms than human beings. Antibacterial and antifungal activities of Ag NPs were tested against *B. cereus*, *S. aureus*, *C. koseri*, *P. aeruginosa* bacteria and *C. albicans* fungus respectively. It has been proposed that Ag NPs penetrate into the bacterial cell and interact with the thiol, hydroxyl and carboxyl groups of the biomolecules present in them, eventually deactivating the vital functions by releasing Ag^+^ ions. The authors have, however, not explained how the Ag^+^ ions were produced. We firmly believe that silver ions must have been produced through a redox mechanism and subsequently complexed with electron donating thiol and phosphate groups inhibiting the cell replication of pathogens. It is well known that silver ions strongly bind with sulfur and oxygen containing electron donor groups in living system and arrest the functioning of vital organs that lead to the death of animal.

Ag NPs synthesized from lingonberry and cranberry juices [241] were tested for their activity against microbes commonly found in food and food products namely, *S. aureus*, *S. typhi*, *L. monocytogenes*, *B. cereus*, *E. coli*, *B. subtillis* and *C. albicans*. They observed that Ag NPs were more effective towards *S. aureus*, *B. subtillis* and *B. cereus*. Antibacterial activity was screened against *B. cereus* and *S. aureus* which produce toxins in food products [243]. A similar study has also been reported by Nanda and Saravanan [168] on other pathogens such as *S. aureus*, *S. epidermidies* and *S. pyogens*. The decrease in antimicrobial effect of Ag NPs against food borne bacteria has been ascribed to low pH or high NaCl content in food. The high concentration of NaCl may increase the toxicity towards bacteria because they may kill them. However, it is concluded that Ag NPs may be used in packaging to prevent infection in food products by microbes.

Zhao and Stevens [273] have studied antimicrobial effects of Ag salts on 12 species of bacteria and showed that they are highly effective against them. It has also been shown [274] that Ag NPs with amphiphilic hyperbranched macro molecules act as antimicrobial coating agents. Kim et al. [275] have thoroughly screened the antimicrobial effect of Ag NPs prepared from AgNO_3_ and NaBH_4_ as reducing agent. They examined the efficacy of a wide range of concentrations of Ag NPs starting from 0.2 to 33 nM. At a concentration of 33 nM of Ag NPs the growth inhibition of *E. coli* and *E. aureus* was almost comparable with the positive control, although at 13.2 nM concentration a significant effect was observed. However, the inhibitory effect of 1.6–6.6 nM of Ag NPs is nearly the same (~ 55% relative to control). It was observed that silver nanoparticle is most effective against *E. coli* and has a mild inhibitory effect on *S. aureus*. However, gold nanoparticles of the same concentration were ineffective against these microbes, although it also belongs to the same group of elements.

Ag NPs synthesized from fungus *Humicola* sp. were investigated for their cytotoxicity on NIH3T3 mouse embryonic fibroblast cell line and MDA-MB-231 human breast carcinoma cell line [224]. In both cell lines, the cell viability declined in a dose-dependent manner. Cytotoxicity of Ag NPs was recorded at a concentration of 250 µg/mL; the cell viability declined by 20 83% in the case of NIH3T3 and 42 18% for MDA-MB-231 cell line at 1000 µg/mL concentration. Very recently [269], it has been investigated that Ag NPs in conjugation with other metals such as TiO_2_@Ag nanoparticles act against leishmaniasis. These nanoparticles along with other drugs for leishmania, like neglumine antimoniate at nontoxic concentrations increase the efficacy of both drugs. This combination of drug led to the inhibition of *L. tropica* amastigotes at a very high rate of 80–95%. Also, it increased the metabolic activities 7–20-fold.

Owaid et al. [237] have produced Ag NPs from aqueous extract of *P. cornucopiae* var. *citrinopileatus* which served both as reducing and stabilizing agent. Their antimicrobial activity was investigated against four pathogenic *Candida* sp. namely *C*. *albicans, C. glabrate, C. krusei* and *C. pseudotropicalis*. Ag NPs at 60 µg/well showed a significant increase in inhibition of *candida* sp. However, pure extract was ineffective against all microbes at 20–40 µg/well. Mechanism of action has been ascribed to the interaction between the positive charge on silver ion and the negative charge on the cell membrane of microorganism [25, 35]. Due to electrostatic attraction between the two the silver ions penetrate into the microbial cell via diffusion leading to their death. Ag NPs synthesized using fungus *Trichoderma viride* were examined for their antimicrobial activity in combination with various antibiotics (ampicillin, kanamycin, erythromycin and chloramphenicol) against both gram positive and gram negative bacteria [234]. Antibacterial activities of antibiotics were increased in the presence of Ag NPs against the tested strains and *P. aeruginosa*. The original aqueous extract of *P. ostreatus* was found to be ineffective against all bacterial strains at 25–75 µg/mL.

Allahverdiyev et al. [276] have reported that the combination of Ag NPs with antibiotics decreases the toxicity toward human cells by reducing the required dosage. Furthermore, these combinations restore the ability of the drug to kill bacteria that have acquired resistance to them [175]. Hence, a separate approach of using Ag NPs synthesized from bacterial strains alone and in combination can act as effective novel antimicrobials to sensitize resistant pathogens. Nevertheless, a study with *E. coli* has demonstrated that the bacteria could become resistant to Ag NPs on its regular exposure for 225 generations through genetic mutations [277]. Thus, a precaution should be taken to avoid the constant exposure of microorganisms against such types of nanoparticles. In addition, treatment with bacterial Ag NPs has shown the cell viability reduction in a dose-dependent manner in HeLa cervical cancer [278, 279], MDA-MB-231breast cancer [280], A549 adenocarcinoma lung cancer [281] and HEP2 [282] cell lines. Ag NPs produced from bacterial strains exhibited cytotoxicity to cancer cells but their impact on normal healthy cells cannot be ignored.

## Mechanism of antibacterial activity

As discussed previously, several reports are available which have shown that Ag NPs are effective against pathogenic organisms namely *B. subtilis*, *Vibrio cholerae*, *E. coli*, *P. aeruginosa*, *S. aureus*, *Syphilis typhus* etc. [10, 11, 109, 145]. Ag NPs with larger surface area provide a better contact with microorganisms [283]. Thus, these particles are capable to penetrate the cell membrane or attach to the bacterial surface based on their size. In addition, they were reported to be highly toxic to the bacterial strains and their antibacterial efficiency is increased by lowering the particle size [284]. Many arguments have been given to explain the mechanism of growth inhibition of microbes by Ag NPs but most convincing is the formation of free radical which has also been supported by the appearance of a peak at 336.33 in the electron spin resonance (ESR) spectrum of Ag NPs [275]. The free radical generation is quite obvious because in a living system they can attack membrane lipids followed by their dissociation, damage and eventually inhibiting the growth of these microbes [285]. It is worth noting that the equal mass of silver Ag NPs and that of Ag ions exhibit identical growth inhibition of *E. coli* and *S. aureus*. In a study, the highly antibacterial activity has been ascribed to the release of silver cation from Ag NPs [173]. The Ag+ permeated into bacteria through the cell wall [286, 287] as a consequence of which the cell wall ruptures leading to denaturation of protein and death. Since Ag ions are positively charged and much smaller than neutral Ag NPs they can easily interact with electron rich biomolecules in the bacterial cell wall containing S or P and N. Some researchers have reported that interaction between the positive charge on Ag NPs and negative charge on the cell membrane of the microorganisms is the key to growth inhibition of the microbes [286, 287]. On the other hand, Sondi et al. [288] have reported that antibacterial activity of Ag NPs toward gram negative bacteria depends on its concentration. The nanoparticles form pits in the cell wall of microbes, get accumulated, and permeate into the bacterial cell leading to their death. It has been reported [289, 290] that Ag free radical formation and antimicrobial property are inter related which has been confirmed by ESR [275]. They claim that such an antimicrobial study included both the positively charged silver ions and negatively charged silver nanoparticles.

The absorption of Ag NPs at 391 nm is the signature of spherical nanoparticles due to their surface plasmon resonance [291]. This absorption spectrum does not undergo any change even when the suspension of Ag NPs is diluted ten times indicating that they are not agglomerated. Besides Ag NPs and silver compounds, there are other inorganic ions which also possess antibacterial properties [241, 287, 292]. It is known that silver ions bind to the protein of the microorganisms preventing their further replication but the organisms also avoid interacting with these ions and produce cysts to become resistant.

Ag NPs may be oxidized to Ag^+^ but cannot be reduced [287, 289]. Silver is known to have 4*d*^10^, 5*s*^1^ outermost electronic configuration and it cannot hold an extra electron to become Ag^−^ anion. Silver salt of sulphathiazine is used in burn therapy to protect the skin from infection by pseudomonas species. Silver is released slowly from the salt which is sufficiently toxic to microorganisms. Since the salt is sparingly soluble the silver acts on the external cell structure. Silver salt and Ag NPs exhibit cytotoxicity against a broad range of microorganisms, although the toxicity depends on the quantum of silver ions released [275].

The monodispersed nanoparticles of uniform size are produced. Graphene oxide exhibits antibacterial activity against *E. coli* [293, 294] but Ag NPs functionalized graphene based material show enhanced antibacterial activity [295, 296]. Graphene oxide is nicely dispersed in polar solvents like water which allows the deposition of nanoparticle for its use in various fields. Antibacterial activity of both Ag NPs and Ag-graphene oxide composite has been tested in a wide range of concentration between 6.25 and 100 µg/mL against both gram positive and gram negative bacteria. It was noticed that both Ag NPs and Ag-graphene oxide composite were more effective against gram positive than gram negative bacterial strains. Ag-graphene oxide is a better growth inhibitor of *S. Typhi*, even at a very low concentration of 6.25 µg/mL, than Ag NPs of the same concentration. However, Ag NPs and Ag-graphene oxide do not show any inhibitory effect against gram positive bacteria, *S. aureus* and *S. epidermis* below 50 µg/mL. It was also noted that graphene oxide alone is ineffective against these bacteria even at a higher concentration of 100 µg/mL [293, 296].

Silver ions released from Ag NPs may penetrate into bacterial cell components such as peptidoglycan, DNA and protein preventing them from further replication [297, 298]. Release of Ag^+^ ions means the oxidation of elemental silver which requires an oxidizing agent.

$$ {\rm Silver}\,\, {\rm nanoparticle} \to {\rm Ag}^+ + {\rm e}^- $$

The organic groups like carbonyl and protein in the bacterial cell wall are electron donors rather than electron acceptors and hence they cannot produce Ag^+^ ions from Ag atoms, nevertheless the Ag^+^ ions are produced which confirms the presence of an oxidizing agent [296, 299]. Ag^+^ ions are thus bonded to the proteins of bacteria and inhibit their vital functions.

Tho et al. [300] have shown that spherical Ag NPs of 2.76–16.62 nm size fabricated from *Nelumbo nucifera* seed extract are highly toxic to Gram negative bacteria. The antibacterial property has been ascribed to the attachment of Ag NPs to the surface of cell membrane disallowing permeation and respiration of the cells.

The outer layer of gram negative bacteria is made up of a lipopolysaccharide layer and the inner layer is composed of a linear polysaccharide chain forming a three-dimensional network with peptides. Ag NPs get accumulated due to attraction between the negative charge on the polysaccharides and weak positive charge on the silver nanoparticles. It stops the cell replication of the microbes.

**Fig. 5:**
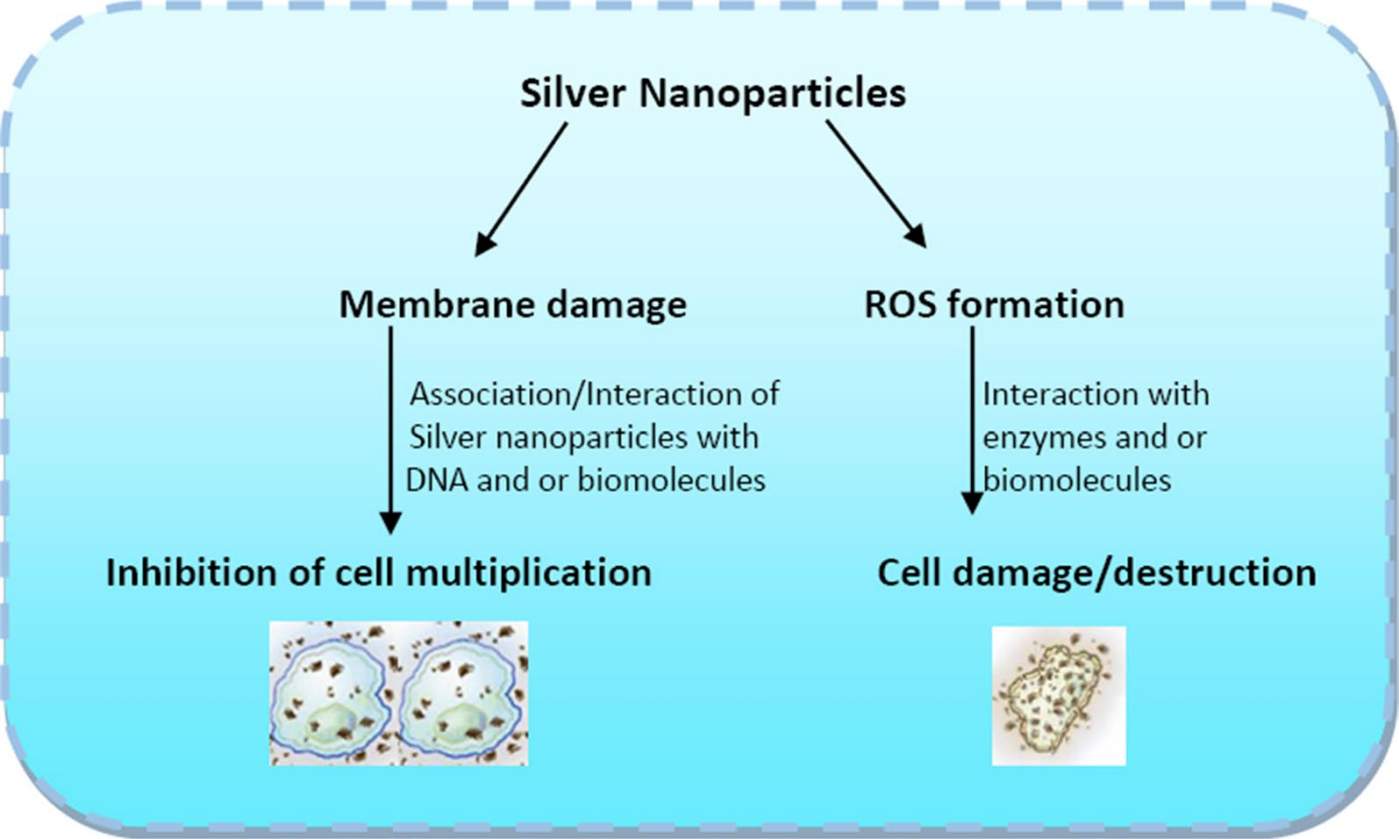
Mechanism of action of silver nanoparticles against bacterial cells

Toxicity by nanoparticles is generally triggered by the formation of free radicals, such as ROS [301, 302]. If the ROS is produced it may cause membrane disruption and disturb the permeability. The mechanism of growth inhibition follows electrostatic interaction, adsorption and penetration of nanoparticles into the bacterial cell wall. Toxicity of nanoparticles also depends on composition, surface modification, intrinsic properties and type of microorganisms [9, 303–306]. For instance, TiO_2_-nanoparticles can increase peroxidation of the lipid membrane disrupting the cell respiration [307]. The biogenic Ag NPs in combination with antibiotics like erythromycin, chloramphenicol, ampicilin and kanamycin enhance the toxicity against gram positive and gram negative bacteria [308, 309]. A possible mechanism is presented in Fig. 5. Besides, Ag NPs are also toxic to nitrifying bacteria [310]. The ROS include superoxide (O_2_^−^), hydroxyl (·OH), peroxy (RCOO·) and hydrogen peroxide (H_2_O_2_). RNS includes nitric oxide (NO·) and nitrogen dioxide (NO_2_^−^) [311, 312]. The cell replication and development of microbes in ROS containing atmosphere will cease to continue. However, this process may be delayed in presence of an antioxidant such as an enzyme or a non-enzymatic component which scavenges the free radicals [313].

## Conclusion

Regardless of the method of fabrication, Ag NPs are used as an antimicrobial agent, electrochemical sensors, biosensors, in medicine, health care, agriculture and biotechnology. They have great bactericidal potential against both gram positive and gram negative pathogens. Since Ag NPs coupled with antibiotics are active against many drug resistant bacteria they can be used as easily accessible medicine for the treatment of several infections. Ag NPs in the drug delivery system, quite often increase the solubility, stability and bio-distribution enhancing their efficiency. In presence of nanoparticles the absorption of medicine increases several times therefore, Ag NPs may be used as a drug delivery system.

Although, the long-term effect of nanoparticles on human health and crops is not clear. A large number of nanoparticles are being explored in many areas of industry technology, biotechnology and agriculture. It is known that various forms of silver from laundry, paints, clothes etc. and biosolids reach the sewage and sludge. It has been reported that nano sized Ag_2_S are formed in the activated sludge as a consequence of the reaction between silver nanoparticles/Ag^+^ ions and the sulfide produced in sewage. It is not possible for Ag NPs in the elemental form to react with evolved H_2_S. Only Ag^+^ ions may react with H_2_S to yield Ag_2_S according to the reaction given below.

$$ 2{\rm AgNO}_3+ {\rm H}_2 {\rm S}  \to  {\rm Ag}_2 {\rm S}+ 2{\rm HNO}_3$$

Ag_2_S or AgNO_3_ may be ionized to give free Ag^+^ ions which inhibit the bacterial growth. Besides many advantages of Ag NPs there are some disadvantages too. They inhibit the growth of nitrifying bacteria, thereby inhibiting the biological nitrogen removal. As little as 1–20 ppm Ag NPs have been reported to be effective against microbes. It is anticipated that Ag NPs may be used as an inexpensive broad spectrum antimicrobial agent to protect plant crops and infections in human beings.
